# Magnetic Resonance Imaging and Serum AFP-L3 and GP-73 in the Diagnosis of Primary Liver Cancer

**DOI:** 10.1155/2022/1192368

**Published:** 2022-03-30

**Authors:** Jun-Jian Yuan, Yan-Dong Xu, Heng Li, Qing-Jin Guo, Guo-Ce Li, Wei Chai, Zhi-Quan Zhang, Ru-Hai Liu

**Affiliations:** ^1^Department of Hepatobiliary Pancreatic Surgery, Cangzhou Central Hospital, Cangzhou, China; ^2^Department of General Surgery, Renqiu Second People's Hospital, Renqiu, China; ^3^Department of Surgery, Cangzhou Heping Hospital, Cangzhou, China; ^4^Department of Colorectal Surgery, Cangzhou Central Hospital, Cangzhou, China; ^5^Nuclear Magnetic Imaging Department, Cangzhou Central Hospital, Cangzhou, China

## Abstract

**Objective:**

To investigate the combined application value of magnetic resonance imaging (MRI) combined with serum alpha-fetoprotein (AFP)-L3 and Golgi protein (GP)-73 in the diagnosis of primary liver cancer.

**Methods:**

The data of 200 patients with suspected liver cancer admitted to our hospital from February 2020 to February 2021 were retrospectively analyzed, and they were randomly divided into an experimental group and a control group, with 100 cases in each group. The experimental group received a combined detection of MRI with serum AFP-L3 and GP-73, and the control group adopted traditional diagnostic methods (spiral computed tomography and serum AFP). The diagnostic yields of the two groups were compared. Surgical resection was performed after the diagnosis of primary liver cancer, and the correlation between the efficacy and combined detection of MRI with serum AFP-L3 and GP-73 levels was analyzed.

**Results:**

The two groups presented comparable general information (*P* >0.05). The surgical results showed 160 cases of primary liver cancer, including 75 cases in the experimental group and 85 cases in the control group, and 40 cases of benign liver lesions. The diagnostic accuracy of the experimental group (73/75, 95%) was significantly higher than that of the control group (76/85, 86%) (*P* < 0.05). The serum levels of AFP-L3, GP-73, and AFP in patients with primary liver cancer were remarkably decreased after surgery (*P* < 0.001). The preoperative and postoperative AFP-L3, GP-73, and AFP levels of patients with primary liver cancer were significantly higher than those of patients with benign liver lesions. The AUC (95% CI) for the combined detection of MRI and serum AFP-L3 and GP-73 levels in patients with surgically confirmed primary liver cancer was 0.747 (0.619-0.874).

**Conclusion:**

MRI combined with serum AFP-L3 and GP-73 presents favorable diagnostic efficiency in the diagnosis of primary liver cancer, which is worthy of clinical application.

## 1. Introduction

Liver cancer is one of the most common malignant tumors with the highest incidence and mortality. The number of new liver cancer cases and deaths in 2020 was approximately 900,000 and over 830,000, with 410,000 new cases and 390,000 deaths in China, which suggests a universally poor prognosis of patients [1–3]. The incidence of liver cancer has increased in recent years, especially that of primary liver cancer in coastal areas of China, and most cases of primary liver cancer are diagnosed at medium to advanced stages, accompanied by metastasis, invasion, apoptosis evasion, and drug resistance, which complicate clinical treatment [4, 5]. Therefore, enhancement of the early diagnosis yield of primary liver cancer is the key to improving the prognosis of patients. Due to the small size and atypical presentation of blood supply in some patients, dynamic contrast-enhanced scanning of benign focal lesions in the liver exhibits close resemblance to liver cancer, leading to difficulties in clinical confirmation of the diagnosis [6, 7]. Accordingly, magnetic resonance imaging (MRI) is considered one of the most optimal diagnostic methods for liver focal lesions.

Compared with ultrasound and spiral computed tomography (CT), MRI multisequence scanning provides better tissue resolution that can distinguish microscopic lesions such as nodules, steatosis, and hemorrhagic necrosis in the liver with a diameter of <1 cm. It facilitates to display the marginal structures of lesions, such as cirrhotic nodules and tumor tissues, and to clarify the hemodynamics of the liver interior [8, 9], with better diagnostic effects than CT for primary liver cancer. In addition to imaging, serum tumor markers are the most common diagnostic means of cancer. Alpha-fetoprotein (AFP) is currently the most common tumor marker in the diagnosis of liver cancer, but clinical experience has revealed its mediocre sensitivity and specificity [10]. A previous study showed that the sensitivity and specificity of AFP for detecting hepatocellular carcinoma was 67.62% and 96.06% [11]. AFP-L3, a member of the AFP family, is aberrantly expressed in cancerous tissue cells supplied by hepatic arteries with favorable sensitivity. In addition, Golgi protein (GP)-73 is also a common diagnostic marker for liver cancer, which presents an extremely low or no expression in hepatocytes and high expression in hepatocytes infected with hepatitis B virus or adenovirus. Previous studies have reported higher sensitivity but lower specificity of GP-73 than AFP assay in the early diagnosis of PLC [12, 13]. Currently, there is no report on the application of MRI combined with AFP-L3 and GP-73 in the diagnosis of primary liver cancer in China. Therefore, this study was designed to evaluate their application value in diagnosing primary liver cancer, to explore the optimal way for the diagnosis and treatment of primary liver cancer.

## 2. Materials and Methods

### 2.1. Study Design

This retrospective study was conducted in our hospital from February 2020 to February 2021.

### 2.2. Inclusion and Exclusion Criteria

Inclusion criteria were as follows: (1) Patients who were suspected liver cancer cases by abdominal ultrasound, physical history, routine CT, and other imaging examinations and (2) patients who met the indications for MRI and spiral CT examinations.

Exclusion criteria were as follows: (1) Patients with factors that may interfere with the detection results, such as other malignant tumors; (2) patients with diseases such as liver metastatic cancer and decompensated liver cirrhosis; and (3) Patients with diseases such as cardiopulmonary insufficiency, renal dysfunction, and mental abnormalities.

### 2.3. General Information of Patients

A total of 200 patients with suspected liver cancer were included in this study, and they were randomly divided into the experimental group and the control group, with 100 cases in each group. There was no statistical difference in the general information of patients in the two groups, as shown in Table 1.

### 2.4. Ethical Considerations

This study were reviewed and approved by Cangzhou Central Hospital, and was in accordance with the principles of the Declaration of Helsinki (2013) [14], and patients and their families were informed of the risks of the study, voluntarily participated in the study, and signed the informed consent form.

### 2.5. Methods

Patients in the experimental group received MRI combined with serum AFP-L3 and GP-73 levels assay, and those in the control group were given traditional diagnostic methods (spiral CT and serum AFP assay). Primary liver cancer was surgically resected immediately after diagnosis.

#### 2.5.1. Detection Method

*(1) Spiral CT Instrument*. Light Speed 16-layer spiral CT (National Instrument Note 20162212460) manufactured by General Electric Company was used for scanning, with a scan thickness of 1 cm. The arterial phase was scanned 25 s after injection of 100 mL iohexol contrast agent (Hunan Hansen Pharmaceutical Co., Ltd., State Drug Administration H20094085) covering the upper abdomen, the venous phase was scanned 65 s after injection, and the delayed phase was scanned 300 s after injection.

*(2) MRI Scanning*. Avantol 1.5 T superconducting MRI produced by SIMENS (Food and Drug Administration Arms Quorum 2014 No. 3280087) was used for liver dynamic contrast-enhanced scanning. The arterial phase was scanned 25 s after injection of 15 mL gadopentetate dimeglumine contrast agent (Beijing Beilu Pharmaceutical Co., Ltd., State Drug Quantifier H20013088) covering the liver, the venous phase was scanned 60 s after injection, and the delayed phase was scanned 200 s after injection.

*(3) Serological Assay*. Serum AFP and GP73 levels were determined using enzyme-linked immunosorbent assay (Beijing Kewei Clinical Diagnostic Reagent Co., Ltd., State Drug Authentication S20060028), and AFP-L3 levels were determined using microcentrifugal column method (YANEN Biotechnology Shenzhen Co., Ltd., Guangdong Shenhua Pharmaceutical Supervision Machinery Authentication 2014 No. 1400008).

#### 2.5.2. Diagnostic Method

The original images of CT scan and MRI were diagnosed by two experienced imaging physicians, to reach a final consistent diagnostic result.

### 2.6. Observation Criteria

#### 2.6.1. General Information

General information contains gender, age, weight, disease type (benign liver lesion or liver cancer, diagnostic criteria for liver cancer: the 2017 edition of the “Diagnostic Code for Primary Liver Cancer” [15]), clinical symptoms, education level, living habits, and medical cost payment method.

#### 2.6.2. Diagnostic Efficacy Analysis of Imaging Examinations

With surgical confirmed results as the gold standard, the differences in imaging characteristics of liver cancer between CT spiral scanning and MRI scanning were compared and analyzed, as well as the sensitivity, specificity, positive likelihood ratio, positive predictive value, and correct diagnostic yield.

#### 2.6.3. Serological Analysis

The differences of serum AFP-L3, GP-73, and AFP levels in patients with benign liver lesions and primary liver cancer were compared, and the differences of serum AFP-L3, GP-73, and AFP levels in patients with primary liver cancer before and after surgery were compared.

#### 2.6.4. Correlation Analysis

ROC curve was used to analyze the correlation between the combined test of MRI, serum AFP-L3, and GP-73 levels and the efficacy.

### 2.7. Statistical Analyses

SPSS 20.0 was used for data analysis and GraphPad Prism 7 (GraphPad Software, San Diego, USA) was used for image rendering. The count data were processed by chi-square test, and the measurement data were analyzed by *t*-test. Differences were considered statistically significant at *P* < 0.05.

## 3. Results

### 3.1. Comparison of General Information

There was no statistical difference between the two groups in terms of general information, such as age and gender ratio (*P* > 0.05), and the surgical results confirmed a total of 160 patients with primary liver cancer and 40 patients with benign liver lesions, as shown in Table 1.

### 3.2. Diagnostic Efficacy Analysis of Imaging Examinations

#### 3.2.1. Spiral CT Scanning

The contrast-enhanced scanning in the hepatic arterial phase showed thickened blood vessels in primary liver cancer lesions, with 40 lesions showing spoke-like enhancement. 70 primary liver cancer lesions in the venous phase showed a gradual weakening of enhancement effect and clear contour, with 15 cases showing isointense performance, i.e., high density in the arterial phase, high and low density in the portal and delayed phases, with rapid enhancement performance.

#### 3.2.2. MRI

Primary liver cancer lesions in the portal and delayed phases mostly showed hypointense or isointense, i.e., high-iso-iso signal and high-iso-low signal, with rapid enhancement performance. Moreover, there were 45 cases of complete circular enhancement, 20 cases without significant enhancement, and 10 cases with incomplete circular enhancement.

The experimental group had 75 patients with primary liver cancer and the control group had 85. The diagnostic accuracy of the experimental group (73/75, 95%) was significantly higher than that of the control group (76/85, 86%) (*P* < 0.05). See Tables 2, 3, 4.

### 3.3. Comparison of Serological Indicators

**Figure 1:**
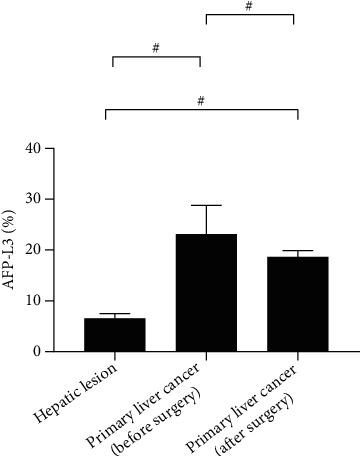
Serum AFP-L3 (^−^x ± s, %). Note: In Figure 1, the abscissa from left to right is patients with benign liver lesions, patients with primary liver cancer (before surgery), and patients with primary liver cancer (after surgery), respectively, and the ordinate is serum AFP-L3 (%). The serum AFP-L3 levels were significantly higher in patients with primary liver cancer (before surgery) than in patients with benign liver lesions and patients with primary liver cancer (after surgery) (23.14 ± 5.65 vs 6.54 ± 0.98, *P* < 0.001). The serum AFP-L3 levels were significantly lower in patients with benign liver lesions than in patients with primary liver cancer (after surgery) (6.54 ± 0.98 vs 18.65 ± 1.22, *P* < 0.001).

**Figure 2:**
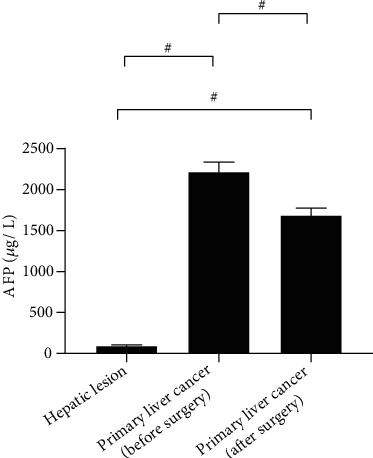
Serum AFP (^−^x ± s, *μ*g/L). Note: In Figure 2, the abscissa from left to right is patients with benign liver lesions, patients with primary liver cancer (before surgery), and patients with primary liver cancer (after surgery), respectively, and the ordinate is serum AFP (*μ*g/L). The serum AFP levels were significantly higher in patients with primary liver cancer (before surgery) than in patients with benign liver lesions and patients with primary liver cancer (after surgery) (2215.65 ± 120.68 vs 95.65 ± 12.55, *P* < 0.001). The serum AFP levels were significantly lower in patients with benign liver lesions than in patients with primary liver cancer (after surgery) (95.65 ± 12.55 vs 1684.65 ± 89.65, *P* < 0.001).

**Figure 3:**
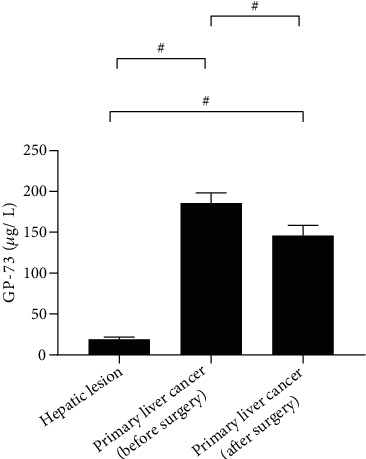
Serum GP-73 (x ± s, *μ*g/L). Note: In Figure 3, the abscissa from left to right is patients with benign liver lesions, patients with primary liver cancer (before surgery), and patients with primary liver cancer (after surgery), and the ordinate is serum GP-73 (*μ*g/L). The serum GP-73 levels were significantly higher in patients with primary liver cancer (before surgery) than in patients with benign liver lesions and patients with primary liver cancer (after surgery) (185.65 ± 12.58 vs 18.98 ± 2.65, *P* < 0.001). The serum GP-73 levels were significantly lower in patients with benign liver lesions than in patients with primary liver cancer (after surgery) (18.98 ± 2.65. vs 145.98 ± 12.50, *P* < 0.001).

**Figure 4:**
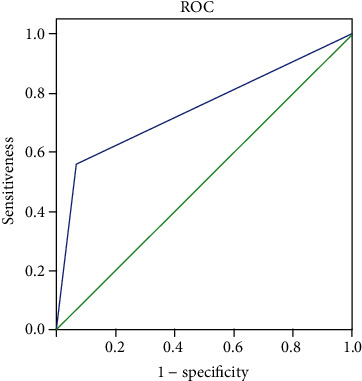
ROC curve analysis of MRI combined with serum AFP-L3 and GP-73 for detecting liver cancer.

The serum levels of AFP-L3, GP-73, and AFP in patients with primary liver cancer were remarkably decreased after surgery (*P* < 0.001). The preoperative and postoperative AFP-L3, GP-73, and AFP levels of patients with primary liver cancer were significantly higher than those of patients with benign liver lesions. See Figures 1–4.

### 3.4. Correlation Analysis

The AUC (95% CI) for the combined detection of MRI and serum AFP-L3 and GP-73 levels in patients with surgically confirmed primary liver cancer was 0.747 (0.619-0.874).

## 4. Discussion

Liver cancer cases in China account for about 58.0% of the world, with mortality and disability rates among the highest levels globally, indicating that liver cancer is a critical medical issue of Chinese society [16]. Currently, surgery is the mainstay of treatment for liver cancer, but most cases are prone to miss the optimal time for surgery due to its insidious symptoms in the early stage, which underlines the significance of early diagnosis and timely surgical treatment. The diagnostic modalities of primary liver cancer mainly include imaging examinations and serological tests, in which imaging examinations contain ultrasound, CT, and MRI. Because the hepatic artery is the main blood supply channel for liver cancer lesions, spiral CT can provide a clinical diagnosis by analyzing the blood supply [17–19], but its display of lesions with a low blood supply level is rather unsatisfactory. Compared with spiral CT, MRI has a stronger soft-tissue resolution, which allows accurate evaluation of the lesion area and the surrounding tissues without the impact of iodine oil deposition. It can be applied in the early examination and postoperative evaluation of liver cancer patients [20, 21]. The results of this study showed that before surgery, primary liver cancer lesions in the portal and delayed phases mostly showed hypointense or isointense, i.e., high-iso-iso and high-iso-low signals, with rapid enhancement performance. There were 45 cases of complete circular enhancement, 20 cases without significant enhancement, and 10 cases with incomplete circular enhancement. After liver cancer surgery, the pathological changes in tumor tissues, unevenly mixed, display different signals on MRI. MRI plain scan can reflect the pathological, size, and morphological changes in the tumor after liver cancer surgery, and contrasted enhanced scan can provide tumor enhancement features [22].

In the present study, of 200 patients with suspected liver cancer, surgical results have confirmed 160 cases of primary liver cancer (75 cases in the experimental group and 85 cases in the control group) and 40 cases of benign liver lesions. Serum tumor marker tests were also employed to further enhance the diagnostic efficiency. AFP is the most common serum marker for liver cancer detection, and an AFP test every 6 months carries a high clinical value for liver cancer screening and prognostic assessment. However, previous research has reported AFP negative cases in liver cancer patients and AFP overexpression in benign liver lesions, which suggests the unavailability of stand-alone diagnosis of early primary liver cancer using serum AFP [23]. With poor effectiveness of spiral CT in detecting microscopic lesions, the combination of the spiral CT and serum AFP only identified 75 positive cases, with a sensitivity of 89.4%, specificity of 66.7%, positive predictive value, positive likelihood ratio, and correct diagnostic rate of 93.8%, 2.685, and 86.0%, respectively; the diagnostic efficiency leaves much to be desired. AFP-L3 is a glycoprotein with abnormal expression in liver cancer cells. It has been reported that AFP-L3 yielded a sensitivity of 36%-96% and a specificity of 89%-94% for liver cancer. In 10%-30% of liver cancers with negative AFP test, the sensitivity and specificity of AFP-L3 were 41.5% and 85.1%, suggesting that AFP-L3 may potentiate the AFP test efficiency [24]. GP-73 is a type 2 Golgi transmembrane glycoprotein that has been reported to yield a higher sensitivity of 77.4-86.3% but a lower specificity than AFP assay in the early diagnosis of primary liver cancer [25]. Herein, the diagnostic accuracy of the experimental group (73/75, 95%) was significantly higher than that of the control group (76/85, 86%).

After surgery, AFP-L3, GP-73, and AFP levels in patients with primary liver cancer were significantly different from those in patients with benign liver lesions (*P* < 0.001), and the AUC (95% CI) for the combined detection of MRI and serum AFP-L3 and GP-73 levels in patients with surgically confirmed primary liver cancer was 0.747 (0.619-0.874), indicating a strong sensitivity of the combined detection. She reported that AFP-L3 can increase the accuracy of MRI in diagnosis, with an AUC (95%CI) = 0.689 (0.584-0.756) [26], confirming a high diagnostic sensitivity of MRI in combination with AFP-L3 in patients with surgically confirmed primary liver cancer, which demonstrates great potential in prognosis evaluation.

In conclusion, MRI combined with serum AFP-L3 and GP-73 presents favorable diagnostic efficiency in the diagnosis of primary liver cancer, which is worthy of clinical application.

## Figures and Tables

**Table 1:** Comparison of general information of patients.

Groups	Experimental group (*n* = 100)	Control group (*n* = 100)	*X* ^2^/*t*	*P*
Gender			0.094	0.760
Male	70	68		
Female	30	32		
Mean age	52.11 ± 2.65	52.34 ± 2.57	0.623	0.534
Mean weight (kg)	54.98 ± 2.78	55.10 ± 2.88	0.300	0.765
Disease types			3.125	0.077
Liver cancer	75	85		
Benign liver lesions	25	15		
Clinical symptoms				
Loss of appetite	65	70	0.570	0.450
Detention	54	50	0.321	0.571
Fatigue	80	85	0.866	0.352
Living habits				
Smoking	55	58	0.183	0.669
Drinking	62	68	0.791	0.374
Education level				
Middle school and below	45	42	0.183	0.669
High school	35	33	0.089	0.765
Junior college and above	20	25	0.717	0.397
Medical payment				
Medical insurance	48	46	0.080	0.777
Business insurance	30	30	≤0.001	1.000
Other	22	24	0.113	0.737

**Table 2:** Diagnostic results of the experimental group.

MRI+ AFP-L3 + GP-73	Pathological examination		Total
	Positive	Negative	
Positive	73	3	76
Negative	2	22	24
Total	75	25	100

**Table 3:** Diagnostic results of the control group.

Spiral CT + AFP	Pathological examination		Total
	Positive	Negative	
Positive	76	5	81
Negative	9	10	19
Total	85	15	100

**Table 4:** Diagnostic efficacy of imaging.

Groups	Sensitivity (%)	Specificity (%)	Positive predictive value (%)	Positive likelihood ratio (%)	Diagnostic yield (%)
MRI + AFP − L3 + GP − 73	97.3 (73/75)	88.0 (22/25)	96.1 (73/76)	8.108	95.0 (95/100)
Spiral CT + AFP	89.4 (76/85)	66.7 (10/15)	93.8 (76/81)	2.685	86.0 (86/100)

## Data Availability

The datasets used during the present study are available from the corresponding author upon reasonable request.
